# Contactless vital signs monitoring in macaques using a mm-wave FMCW radar

**DOI:** 10.1038/s41598-024-63994-w

**Published:** 2024-06-15

**Authors:** Jiajin Zhang, Renjie Hu, Lichang Chen, Yu Gao, Dong-Dong Wu

**Affiliations:** 1 State Key Laboratory of Genetic Resources and Evolution, Kunming Natural History Museum of Zoology, Kunming Institute of Zoology, Chinese Academy of Sciences, Kunming, 650201 China; 2 College of Big Data, Yunnan Agricultural University, Kunming, 650201 China; 3 National Resource Center for Non-Human Primates, Kunming Primate Research Center, and National Research Facility for Phenotypic & Genetic Analysis of Model Animals (Primate Facility), Kunming Institute of Zoology, Chinese Academy of Sciences, Kunming, 650107 Yunnan China; 4 Center for Excellence in Animal Evolution and Genetics, Chinese Academy of Sciences, Kunming, 650201 China

**Keywords:** Non-human primate, Contactless monitoring, Vital signs, Macaque, FMCW radar, Animal welfare, Biological techniques, Engineering, Biomedical engineering, Electrical and electronic engineering

## Abstract

Heart rate (HR) and respiration rate (RR) play an important role in the study of complex behaviors and their physiological correlations in non-human primates (NHPs). However, collecting HR and RR information is often challenging, involving either invasive implants or tedious behavioral training, and there are currently few established simple and non-invasive techniques for HR and RR measurement in NHPs owing to their stress response or indocility. In this study, we employed a frequency-modulated continuous wave (FMCW) radar to design a novel contactless HR and RR monitoring system. The designed system can estimate HR and RR in real time by placing the FMCW radar on the cage and facing the chest of both awake and anesthetized macaques, the NHP investigated in this study. Experimental results show that the proposed method outperforms existing methods, with averaged absolute errors between the reference monitor and radar estimates of 0.77 beats per minute (bpm) and 1.29 respirations per minute (rpm) for HR and RR, respectively. In summary, we believe that the proposed non-invasive and contactless estimation method could be generalized as a HR and RR monitoring tool for NHPs. Furthermore, after modifying the radar signal-processing algorithms, it also shows promise for applications in other experimental animals for animal welfare, behavioral, neurological, and ethological research.

## Introduction

Heart rate (HR) and respiration rate (RR) are critical tools for assessing both physiological and psychological states in animals^1–4^. Among non-human primates (NHPs), rhesus macaques are critically important as a NHP model of human health and disease, including in neuroscience, cognition, and behavioral studies^5^. Nevertheless, the application of vital signs monitoring in NHPs has been limited to date^6^. Very few methods are currently available for easy, reliable, and non-invasive vital signs monitoring in untrained NHPs^7^.

Traditionally, the most common contact sensing tool for HR and RR measurement is an electrocardiogram (ECG)^8^. For example, ECG sensors were employed in NHPs, e.g., ECGs from monkeys under anesthesia^9,10^, an implanted sensor^11,12^, wearable jacket systems for non-sedated monkeys^13^. However, this approach may affect the monkey’s physiological state and raise ethical concerns. Owing to the indocility of NHPs, measuring their physiological parameters using conventional contact measurement techniques has become a challenging task^4^.

In recent years, non-invasive sensing techniques have been developed for monitoring NHP physiological parameters. Photoplethysmography (PPG) is a popular HR monitoring optical technology. However, the body surface condition causes intricacy in PPG, which limits its application in animals^14^. Hence, imaging photoplethysmography (IPPG) has been proposed as a remote and contactless alternative to conventional PPG in humans. Via IPPG, the HR of rhesus monkeys from facial videos has been accurately estimated^15^ and the possibility of obtaining vital signs from anesthetized animals was demonstrated^16,17^. Additionally, Froesel et al.^18^ proposed an automated video-based method using Eulerian video magnification (EVM) and wavelet transformation to monitor the HR of macaques.

However, in daily living scenarios within a cage containing bars, meshes, and other objects (e.g., water bottles and food boxes), the robustness of video-based methods for HR estimation in macaques is limited for practical applications. These methods are easily affected by ambient lighting, video quality, occlusions, motion artifacts, and other environmental factors.

With the rapid development of contactless biomedical sensing technology, radio-frequency (RF) radar technology, which detects vital signs using reflected radio signals to infer micro-displacements in the cardiopulmonary system, has become increasingly prevalent in recent years. For example, an ultra-wideband (UWB) radar was explored to extract vital signs from animals using the range of motion on the body surface caused by cardiopulmonary activity in anesthetized dogs and cats^19^. Furthermore, frequency-modulated continuous wave (FMCW) radar technology was used to detect human vital signs; the results proved that the FMCW radar could precisely measure slight vibrations, such as HR and RR^20–25^.

In this paper, we propose an FMCW-radar-based contactless monitoring scheme for the vital signs of awake and anesthetized macaques. The proposed scheme offers several advantages over UWB radars, including improved accuracy in terms of angle, range, and velocity and better resolution. Furthermore, it does not require a special environment and only requires an FMCW radar device mounted outside the cage. This method provides a low-cost, scalable solution for accurately tracking the HR and RR signals of macaques in their daily living environment.

## Materials and methods

### Animals and ethical considerations

Thirteen laboratory-housed macaques (*Macaca mulatta* and *Macaca fascicularis*) participated in this study. They were aged between 2–3 and 6–7 years old, respectively. Details regarding the macaques are listed in Table 1. To enhance our measure system's robustness and adaptability, we selected macaques as subjects with two species, genotypes (both genetically and non-genetically edited) and states (awake and anesthetized).

**Table 1:** Specimen Information.

Macaque ID	Species	Genotype	Sex	State	Age	Weight kg
16104	*Macaca fascicularis*	Non-genetically modified	Female	Awake	6	3.5
152024	*Macaca fascicularis*	Genetically modified	Female	Awake	7	5.0
152030	*Macaca fascicularis*	Genetically modified	Female	Awake	7	3.6
152032	*Macaca fascicularis*	Genetically modified	Female	Awake	7	3.6
15092	*Macaca mulatta*	Non-genetically modified	Female	Awake	7	5.7
151010	*Macaca mulatta*	Genetically modified	Female	Awake	7	6.4
151007	*Macaca mulatta*	Genetically modified	Male	Awake	7	6.7
151009	*Macaca mulatta*	Genetically modified	Male	Awake	7	6.8
19080	*Macaca mulatta*	Non-genetically modified	Female	Anesthetized	3	6.6
19092	*Macaca mulatta*	Non-genetically modified	Female	Anesthetized	3	5.2
20005	*Macaca mulatta*	Non-genetically modified	Male	Anesthetized	2	4.5
20042	*Macaca mulatta*	Non-genetically modified	Female	Anesthetized	2	4.2
20313	*Macaca mulatta*	Non-genetically modified	Male	Anesthetized	2	3.6

The macaques were acquired from the Kunming Primate Research Center, Kunming Institute of Zoology, Chinese Academy of Sciences, Kunming, Yunnan, China. The Kunming Primate Research Center animal facility is internationally accredited by the Association for Assessment and Accreditation of Laboratory Animal Care (AAALAC).

This study was approved by the Institutional Animal Care and Use Committee of the Kunming Institute of Zoology (approval number: IACUC-PE-2022–11-003). All animals were handled in strict accordance with good animal practices as defined by the relevant national and local animal welfare entities. All experiments were performed in accordance with relevant guidelines and regulations and authors complied with the ARRIVE guidelines.

### FMCW radar working principle

**Figure 1:**
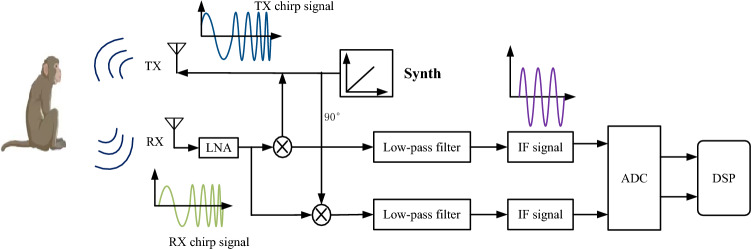
FMCW radar block diagram.

Vital signs detection via FMCW radar functions based on the phase term of the signal reflected by the macaque body. Figure 1 shows the block diagram of a typical FMCW radar system.

The FMCW radar system consists of a transmitting (TX), RF synthesizer, receiving (RX), clock, analog-to-digital converter (ADC), digital circuit, single-chip microcomputer (MCU), and digital signal processor (DSP). The FMCW radar periodically transmits a chirped signal generated by the synthesizer with a frequency that increases linearly with time through a transmitting antenna. Each transmitting chain has independent phase and amplitude control. The RF synthesizer creates a desired frequency, such as a chirped signal that changes over time.

The signal received by the receiving antenna is amplified using a low-noise amplifier (LNA) and correlated with the local chirp of the mixer. After low-pass filtering, a sinusoidal signal containing the instantaneous frequency difference between the transmitting and receiving chirps is obtained, which is called a beat signal or an intermediate frequency (IF) signal.

**Figure 2:**
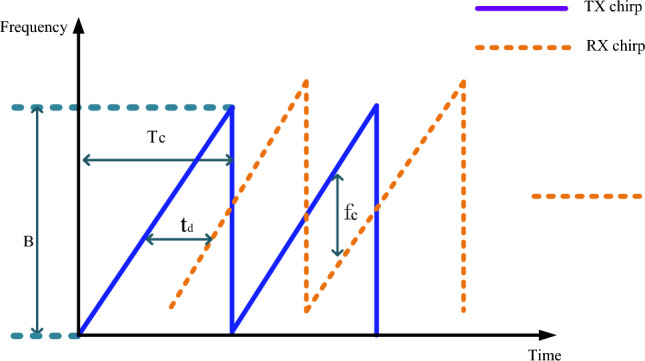
Transmitted and received chirp signals.

Finally, the ADC samples the IF signal for subsequent signal processing. With this instantaneous signal, the instantaneous frequency difference can be translated into the instantaneous distance between the radar and target. The transmitted signal is usually a triangular waveform or sawtooth waveform, which is adopted in this study, and its specific mode is shown in Fig. 2.

The transmitted FMCW signal can be expressed as^26^:

1 $$xT\left( t \right) = A_{TX}COS\left( {2\pi f_{c}t + \pi \frac{B}{Tc}t^{2} + \theta \left( t \right)} \right).$$

where ${f}_{c}$ is the initial frequency of the chirp signal,$B$ is the bandwidth, ${A}_{TX}$ is the amplitude of the transmitted signal, $\theta \left(t\right)$ is the phase noise, $Tc$ is the width of the chirp signal pulse, and $B/ Tc$ is the slope of the chirp signal.

Let $R(t)$ be the motion displacement of the macaque’s body and ${d}_{0}$ the distance from the FMCW radar sensor to the macaque, then the distance from the macaque’s chest to the radar is $x(t)= R(t)+{d}_{0}$, and the time delay is ${t}_{d}= 2x(t)/c$, where $c$ is the velocity of light. Then, the received signal is

2 $$xR\left( t \right) = A_{RX}\left( {COS\left( {2\pi f_{c}\left( {t - t_{d}} \right)} \right) + \pi \frac{B}{Tc}\left( {t - t_{d}} \right)^{2} + \theta \left( {t - t_{d}} \right)} \right).$$

To measure these small displacements, with smaller wavelengths yielding a better displacement sensitivity, $\Delta \emptyset b$ corresponds to the change in phase when the macaque moves a distance $\Delta R$.

For example, at a FMCW radar wavelength of $\lambda$ = 4 mm, when we have displacements as small as $\Delta R$ = 1 mm, the corresponding phase change is $\emptyset b = \pi$. We measured the change in phase of the FMCW radar signal with time at the target range bin as

3 $$\Delta \phi b=\frac{4\pi }{\lambda }\Delta R.$$

Furthermore, we measured the change in phase of the received signal from the macaque at range $R$ after mixing, and the filtering is given by

4 $$xR\left( t \right).xT\left( t \right) \approx e^{{j\left( {4\pi \frac{BR}{{cT}} + \frac{4\pi }{\lambda }\Delta R} \right)}} = e^{{j\left( {f_{c}t + \phi b} \right)}} .$$

For a single object, the beat signal $b(t)$ is sinusoidal and has both frequency $fc$ and phase $\emptyset b$. The phase can be measured by taking a fast Fourier transform (FFT) of the beat signal $b(t)$ and computing the phase in the object range bin. Then, the received signal is

5 $$b\left( t \right) = e^{{j\left( {f_{c}t + \phi b} \right)}} .$$

The echo and transmission signals are mixed by two orthogonal channels and passed through a low-pass filter to obtain the IF signal ${S}_{IF}(t)$ as

6 $$S_{IF}\left( t \right) = A_{RX}A_{TX}\exp \left( {j\left( {2\pi \left[ {\frac{B}{Tc}t_{d}} \right]t + 2\pi f_{c}t + \pi \frac{B}{Tc}{t_{d}}^{2} + \Delta \theta \left( t \right)} \right)} \right).$$

### Process flow for vital signs detection

**Figure 3:**
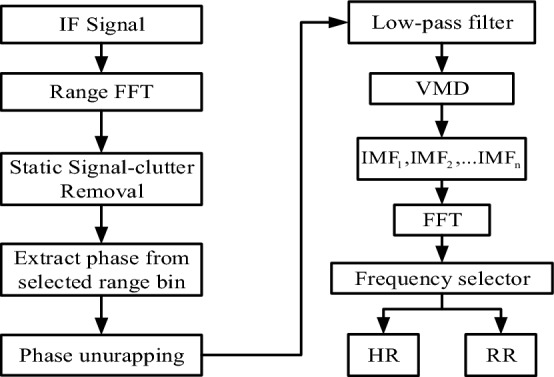
Proposed processing flow for macaque vital signs measurement.

Figure 3 shows the macaque heartbeat and respiratory signal detection process. The process comprises four stages: (1) Target positioning according to the spectrum to obtain the range bin representing the macaque body; (2) Phase extraction from the range bin representing the macaque body in each frame and phase unwrapping to obtain the vital signs signal; (3) Vital sign signal decomposition to obtain the breathing and heartbeat signals; and (4) Respiration and heartbeat rate estimation.

After the IF signal is sampled by the ADC, the FMCW radar data acquisition board exports the sampled data to form a two-dimensional matrix. The sampled data in each chirp constitute the columns of the matrix, while the matrix rows are composed of different frames. It should be noted that we set only one chirp for each frame.

The sampled data were pre-processed mainly through static signal-clutter removal to eliminate environmental background interference for the position information of the measured target. FFT is successively applied to the column vectors of the matrix to obtain the precise range window of the measured target, and then we further extract the phase of each frame in the range window.

To obtain real displacement profiles, the phase data must be unwrapped. The phase information can be used to determine the target's vital signs.

After passing through a low-pass filter, the vital signs are used as the input signal for variational modal decomposition (VMD). By applying the VMD algorithm to the received data through windowing, filtering, and denoising, macaque intrinsic mode functions (IMFs) can be acquired, and they are transformed via FFT and a frequency selector to ultimately obtain the HR and RR. Details of each step are described in the following sections.

### Range FFT and static signal-clutter removal

The target range is a crucial factor in the FMCW radar system. By utilizing the range information, we can find the range bin to extract the signal caused by the movement of the macaque’s chest wall.

First, FFT was applied on the two-dimensional matrix formed by sampling the IF signal to obtain the range slow-time matrix. Owing to the complex background in the field, the target range bin selection is affected by the reflected static clutter signals, which also introduce some phase noise. Thus, the signal interference of the static clutter should be removed.

A simple mean remover was used in this study by removing the average slow-time signal on the range-slow-time matrix from each of its range bins to remove the background noise and obtain the movement information of the target macaque.

### Target range profile reconstruction and phase extraction

The steps to detect the vital signs of the target macaque are as follows:

Step 1: Beat signals sampled ${S}_{b}$[$n$] after the ADC are a two-dimensional matrix ${S}_{RD}$[$m$,$n$], where $m=1, 2, . . ., M$ and $n = 1, 2, . . ., N$ being the number of slow- and fast-time samples, respectively.

Step 2: Apply FFT to each row of ${S}_{RD}$[$m, n$] to obtain the range-profile matrix ${S}_{RP}$[$m, p$], where $p = 1, 2, . . ., N$. Each row of ${S}_{RP}$[$m, p$] is a range profile.

Step 3: Determine the value of $p$ corresponding to the range bin in which the macaque is located. By calculating the variance of each row of ${S}_{RP}$[$m, p$], the range bin with the maximum variance is selected as the target range. Then, we can obtain the signal $S\left[n\right]$ corresponding to the target range bin.

Step 4: The phase information $\Phi [n]$ can be extracted from signal $S[n]$ and set $\Phi \in [-\pi ,\pi ]$. One procedure for unwrapping phase signal $\Phi [n]$ is most easily explained in the following pseudo-code:

$$\begin{aligned} & {\text{While}}\;\;\Phi \left[ {n + 1} \right] - \Phi \left[ n \right] \ge \pi \;\; {\text{do}} \\ & \quad \Phi \left[ n \right] = \Phi \left[ n \right] + 2\pi \\ & {\text{End while}} \\ & \Phi \left[ {n + 1} \right] = \Phi \left[ n \right] \\ \end{aligned}$$

Step 5: Next, considering that the respiratory and heartbeat rates of normal awake macaques are 30–50 respirations per minute (rpm) and 120–180 beats per minute (bpm), respectively^27^, the rpm and bpm of anesthetized macaques are lower than those of awake macaques. Consequently, the phase-unwrapping signal is filtered using a low-pass filter into a heartbeat frequency spectrum of 0.1–3.0 Hz and a respiratory frequency spectrum of 0.1–1.0 Hz.

### Vital signal decomposition with VMD method

As macaque cardiopulmonary systems, like humans, have a certain physiological coupling relationship, the higher harmonics of the respiratory signal may overlap with the heartbeat signal.

To effectively separate the respiratory and heartbeat signals contained in the macaque physical signs information, a signal decomposition method was used to decompose them using the VMD algorithm. The decomposition process decomposes the signal into several sub-signals with limited bandwidth, which are adaptive. The non-recursive quasi-orthogonal decomposition method is essentially the process of solving variational problems, and the constraint condition is that the sum of the IMFs is equal to the input signal $f$.

First, assuming that the $k$ IMFs obtained via decomposition have a certain center frequency and bandwidth, the center frequency and bandwidth of the IMFs are continuously updated during the decomposition process such that the sum of the estimated bandwidth of all IMFs is minimized. This model can be expressed as follows^28^:

7 $$\mathop {\min }\limits_{{\left\{ {u_{k} } \right\},\left\{ {\omega_{k} } \right\}}} \left\{ {\sum\limits_{k = 1}^{K} {\parallel \partial t\left[ {\left( {\delta \left( t \right) + \frac{j}{\pi t}} \right)*u_{k} \left( t \right)} \right]e^{ - j\omega kt} \parallel }_{2}^{2} } \right\}s.t.\sum\limits_{k = 1}^{K} {u_{k} = f}$$

where $K$ is the number of variational intrinsic mode function (VIMFs), while $f$ is the input signal. {${u}_{K}$} = {${u}_{1}, {u}_{2}, \cdot \cdot \cdot , {u}_{K}$} and {${\omega }_{K}$} = {${\omega }_{1},{\omega }_{2}, \cdot \cdot \cdot ,{\omega }_{K}$} are the shorthand notations for the set of all modes and their center frequencies, respectively. Equation (9) can be solved by introducing a quadratic penalty and Lagrangian multipliers. The augmented Lagrangian is expressed as follows^28,29^:

8 $$L\left( {\left\{ {u_{k} } \right\},\left\{ {\omega_{k} } \right\},\lambda } \right) = \mathop {\alpha \sum\limits_{k = 1}^{K} \parallel \partial_{t} \left[ {\left( {\delta \left( t \right) + \frac{j}{\pi t}} \right)*u_{k} \left( t \right)} \right]e^{ - j\omega kt} \parallel }\nolimits_{2}^{2} + \parallel f\left( t \right) - \mathop {\sum\limits_{k = 1}^{K} {u_{k} \left( t \right)\parallel } }\nolimits_{2}^{2} + \left\langle {\lambda \left( t \right),f\left( t \right) - \sum\limits_{k = 1}^{K} {u_{k} \left( t \right)} } \right\rangle$$

where $\alpha$ denotes the balancing parameter of the data-fidelity constraint. Equation (9) is then solved using the alternate direction method of multipliers^30^. All modes gained from solutions in the spectral domain are expressed as follows:

9 $$\hat{u}_{k} \left( \omega \right) = \frac{{\hat{f}\left( \omega \right) - \sum\limits_{i \ne k} {\hat{u}_{i} \left( \omega \right)} + \frac{{\hat{\lambda }\left( \omega \right)}}{2}}}{{1 + 2\alpha \left( {\omega - \omega_{k} } \right)^{2} }}$$

where the ${\omega }_{k}$ is computed at the center of gravity of the power spectrum of the corresponding mode. Thus, Wiener filtering was embedded into the VMD algorithm to make it more robust to sampling and noise.

10 $$\omega_{k} = \frac{{\int_{0}^{\infty } {\omega \left| {\hat{u}_{k} } \right|}^{2} d\omega }}{{\int_{0}^{\infty } {\left| {\hat{u}_{k} } \right|^{2} d\omega } }}$$

A detailed description of the complete VMD algorithm can be found in Dragomiretskiy & Zosso (2013)^28^. After passing through a low-pass filter, the vital signs are used as the input signal of the VMD. An appropriate decomposition level $k$ is set to reduce the aliasing phenomenon of the decomposed natural mode components. The value of $k$ was selected based on actual measured signals.

The decomposed natural mode components are transformed via 1024-point FFT to obtain the spectrum information. Due to the macaque respiratory and heartbeat frequency ranges (0.5–0.8 and 2–3 Hz, respectively), the frequency selector can be used to extract the final RR and HR successively.

### Experimental setup

**Figure 4:**
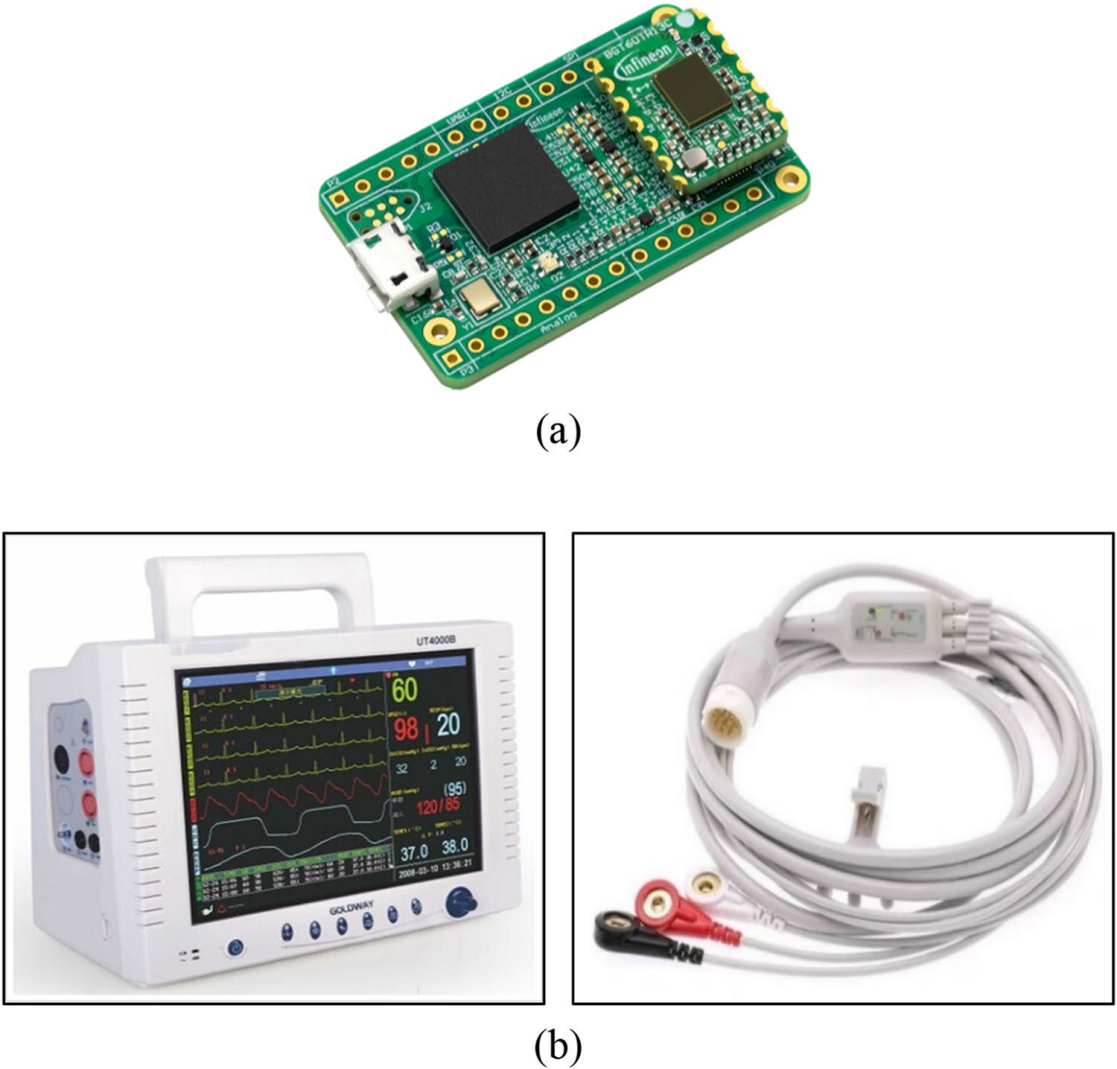
(**a**) BGT60TR13C radar and (**b**) reference ECG device (Gold way Ut4000b) used in this study.

The BGT60TR13C FMCW radar from Infineon Technologies AG, which is equipped with three receiving antennas and one transmitting antenna, was used for data acquisition. Its operating frequency ranges from 57 to 64 GHz, with an adjustable chirp duration^31^. The BGT60TR13C radar is shown in Fig. 4a. In addition, the radar parameters during the experiment were set as listed in Table 2.

**Table 2:** Radar parameters.

BGT60TR13C System Parameter	Value
Gain range	50 db
Bandwidth	3 GHz
Output power	5 dBm
Chirp duration	64 us
Frequency range	63.5 GHz
Starting frequency	58 GHz
ADC sampling rate	4 Msps
Slow time axis sampling	20 Hz

As shown in Fig. 4b, an electrode-based ECG reference device provided vital signs as ground truth to analyze the radar performance. The ECG was used to capture the vital signs of macaques, which were acquired synchronously using the BGT60TR13C radar equipment.

We evaluated the robustness of the proposed method in two states: awake and anesthetized macaques. Eight awake macaques were recorded in the first state. For the latter, five anesthetized macaques were used.

#### Experimental setup for awake macaques

The macaques engaged in the current study had previously participated as subjects in ECG monitoring experiments, boasting a wealth of experience in this experimental setting. For the current trial, we trained eight macaques to perform a sit-in behavioral task for approximately 60 s and return on command. The macaques were individually moved from their home cages to the testing laboratory and calmly seated in a NHP box chair after receiving training with positive reinforcement.

On the test day, the macaque arms and legs were gently restrained with leather straps. The macaques were maintained in the chair for at least one minute and periodically given food rewards for sitting calmly and facing the radar under the breeder’s commands. Thus, all the macaques’ heartbeat and respiratory rates stabilized by the end of the habituation session. This allowed HR and RR recordings to be performed effectively.

**Figure 5:**
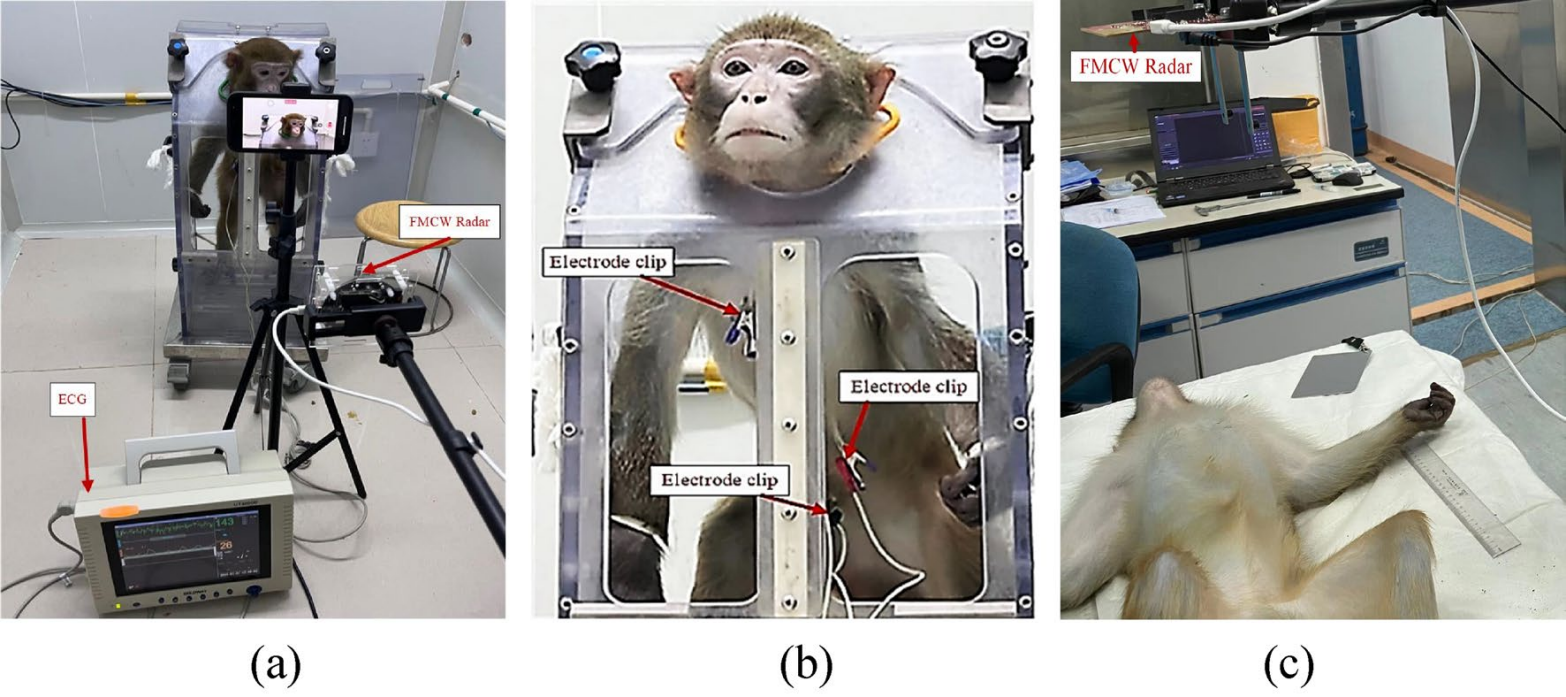
Experimental setup for (**a**,**b**) awakes and (**c**)anesthetized macaques.

The experimental setup for awake macaques is shown in Fig. 5a,b. The radar antennas were positioned in front of the macaques’ chest, and the electrocardiogram electrodes were positioned on their chest and stomach. The macaques HR and RR were measured simultaneously via radar and ECG for 60 s.

#### Experimental setup for anesthetized macaques

In order to further verify the accuracy and reliability of our method, the macaques marked 19080, 19092, 20005, 200402, and 20313 were anesthetized during measurement, as shown in Table 1.

As shown in Fig. 5c, the macaques were lightly anesthetized with a ketamine hydrochloride injection (0.5 ml) to avoid body movements. During radar and ECG acquisitions, the macaques were constantly resting on the surgery table with minimal movement. The radar was positioned toward the macaques’ chest at a proper distance with an angle of approximately 90°. A vital signs measurement period of approximately one minute was chosen.

## Institutional review board statement

This study was approved by the Institutional Animal Care and Use Committee of the Kunming Institute of Zoology (approval number: IACUC-PE-2022–11-003). All animals were handled in strict accordance with good animal practices as defined by the relevant national and local animal welfare entities.

## Informed consent

Informed consent was obtained from all subjects involved in the study.

## Experimental results

### Target detection

According to the FMCW radar signal characteristics, the larger the distance between the macaque body and the radar, the lower the signal-to-noise ratio (SNR) of the vital sign’s waveform. To achieve the best performance of the proposed method, experiments were performed under different ranges. The eight awake macaques were seated in a stationary position at different distances from the radar, namely, at 0.3, 0.6, and 0.9 m, respectively. The HR and RR values were recorded at each distance via the ECG and radar. We found that the range for macaque monitoring should be restricted to 0.6 m.

**Figure 6:**
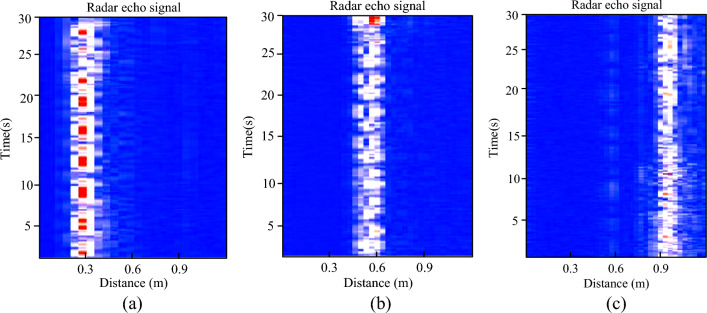
Macaque radar echo signal at a distance of (**a**) 0.3, (**b**) 0.6, and (**c**) 0.9 m from the radar.

As shown in Fig. 6, for the macaque marked as 152024, radar echo signals were collected in the three scenarios and computed for an acquisition time of 30 s.

For the 0.3 and 0.9 m distances, many additional small harmonics appeared in the echo signal, as shown in Fig. 6a,c, respectively^21,25,32^. The radar echo signal of the target is shown in Fig. 6b, in which the distance information of the macaque and its periodic vital signs is obvious. The respiratory frequency and heartbeat could be successfully retrieved.

### HR and RR signal separation and reconstruction

**Figure 7:**
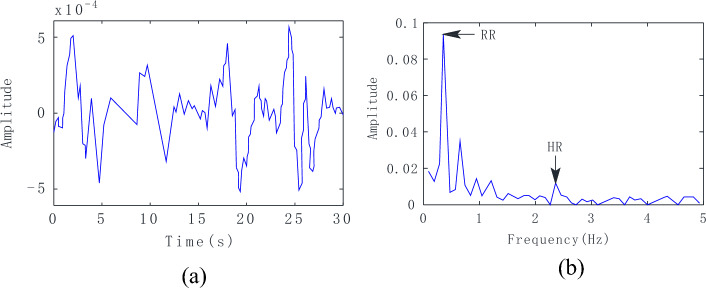
Signal results. (**a**) Radar echo signal; (**b**) Spectrum of the radar echo signal.

After the background removal process, the radar echo signal waveform of one awake macaque is shown in Fig. 7a, where obvious respiratory waveforms and weaker heartbeat pulsations can be observed. By applying the VMD algorithm, the signal spectrogram was obtained, as shown in Fig. 7b, from which the macaque HR and RR could be directly obtained.

Although macaque heartbeat signals can be conveniently obtained using filters for known heartbeat frequencies, it is difficult to choose an appropriate filtering range for practical applications. An 8-s signal was selected for processing to better reflect changes in HR and RR. Referring to^33^, the input signal in an 8 s-time window is through to the bandpass filter of which cut frequencies are from 0.4 to 5 Hz, covering the normal macaque heart rate range (about 0.5–3 Hz). Note that an 8-s time window is an appropriate size for heartbeat detection and can achieve high spectral resolution.

**Figure 8:**
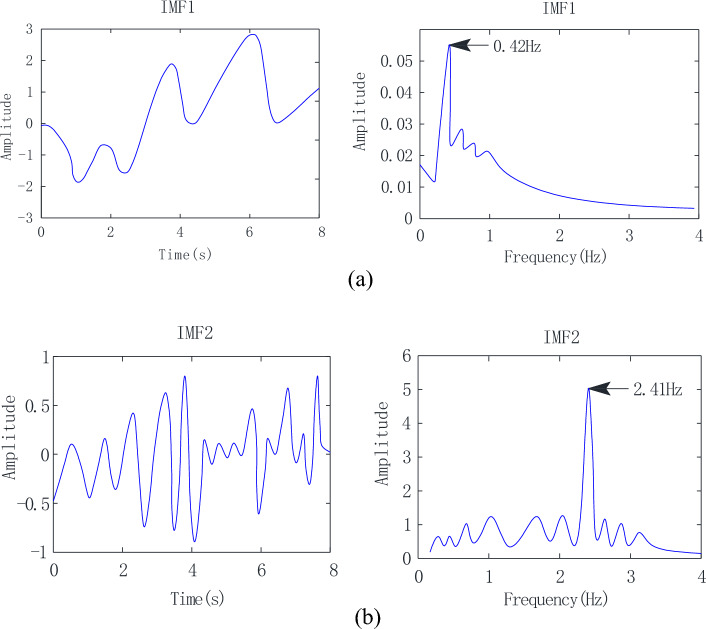
Results after applying the VMD algorithm. (**a**) IMF1 and (**b**) IMF2 waveforms and spectra.

As shown in Fig. 8a, only three respiratory cycles are included in the 8-s signal, with a respiratory frequency of 0.42 Hz obtained via FFT. Figure 8b displays the IMF2 waveform and spectrum, as well as the regular heartbeat signal cycle. The heartbeat frequency obtained via FFT is 2.41 Hz.

The pre-processed data are given as the input parameters of the VMD algorithm. The number of modes was set to four. The balancing parameter and tolerance were set to 10,000 and 10^−6^, respectively. Subsequently, real-time decomposition results could be obtained. According to the frequency range, IMF1 is the RR signal component, while IMF2 is the HR signal component.

**Figure 9:**
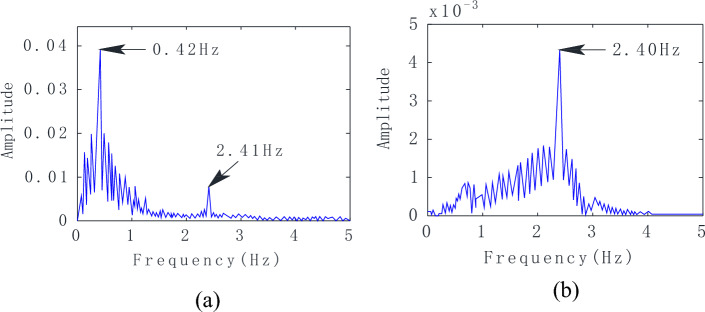
Spectrum results. (**a**) Spectrum of the macaque's vital signs; (**b**) Frequency of the macaque's heartbeat.

By applying FFT, we can directly calculate the HR and RR of the target. The macaque vital signs spectrum is shown in Fig. 9a. In the figure, we can see that the respiratory frequency of the macaques obtained via FFT was 0.42 Hz. Furthermore, as shown in Fig. 9b, the heartbeat frequency obtained via FFT was 2.40 Hz.

### Statistical analysis

The data collected from the 13 macaques were analyzed statistically to characterize the FMCW radar performance and to determine whether the proposed system could be used effectively for macaque vital signs monitoring. The data for all macaques were measured by radar with an acquisition time of 30 s.

First, we summarized the average HR of the macaques from the ECG and radar, and analyzed the data of the awake and anesthetized macaques, as shown in Table 3, where Abs. Error and Rel. Error are the absolute and relative errors, respectively.

**Table 3:** HR average values of macaques detected via ECG and radar.

Macaque ID	State	ECG	Radar	Abs. error	Rel. error (%)
16104	Awake	147.50	148.67	1.17	0.79
152024	Awake	145.83	144.50	1.33	0.91
152030	Awake	138.83	139.00	0.17	0.12
152032	Awake	150.50	150.17	0.33	0.22
15092	Awake	147.00	145.50	1.50	1.02
151010	Awake	160.00	159.67	0.33	0.21
151007	Awake	130.50	131.33	0.83	0.64
151009	Awake	128.83	130.17	1.34	1.04
19080	Anesthetized	86.34	86.50	0.16	0.19
19092	Anesthetized	101.67	100.83	0.84	0.83
20005	Anesthetized	85.50	84.00	1.50	1.75
20042	Anesthetized	78.00	77.83	0.17	0.22
20313	Anesthetized	87.00	86.67	0.33	0.38

Table 3 shows the performance of the proposed system in estimating the HR of awake and anesthetized macaques. For the ECG measurements, the average HR of the awake macaques was 143.62 ± 9.75 bpm, while that of the anesthetized macaques was 87.70 ± 7.70 bpm. For the radar measurements, the average HR of the awake macaques was 143.63 ± 9.24 bpm, while that of the anesthetized macaques was 87.17 ± 7.54 bpm.

A comparison between the ECG and radar measurements showed that the mean absolute errors were 0.88 and 0.60 bpm for awake and anesthetized macaques, respectively; the mean relative errors were 0.62% and 3.37% for awake and anesthetized macaques, respectively.

**Figure 10:**
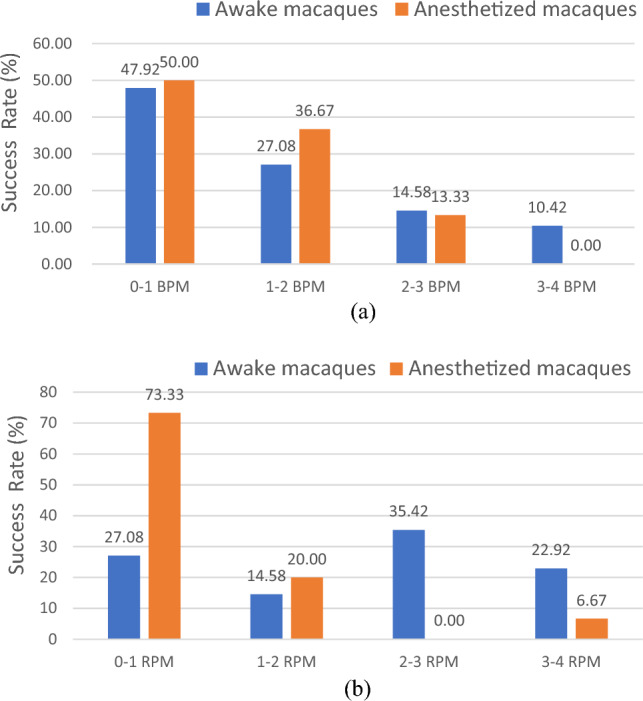
Success rate of the radar measurements for (**a**) HR and (**b**) RR.

Figure 10a shows the measurement success rates. For the awake macaques, the HR error remained under 4 bpm in 100% of the measurements, and 89.58%, 75.00%, and 47.92% at 3, 2, and 1 bpm, respectively. For the anesthetized macaques, all measurement errors remained under 3 bpm, and under 2 and 1 bpm for 86.67% and 50%, respectively.

**Figure 11:**
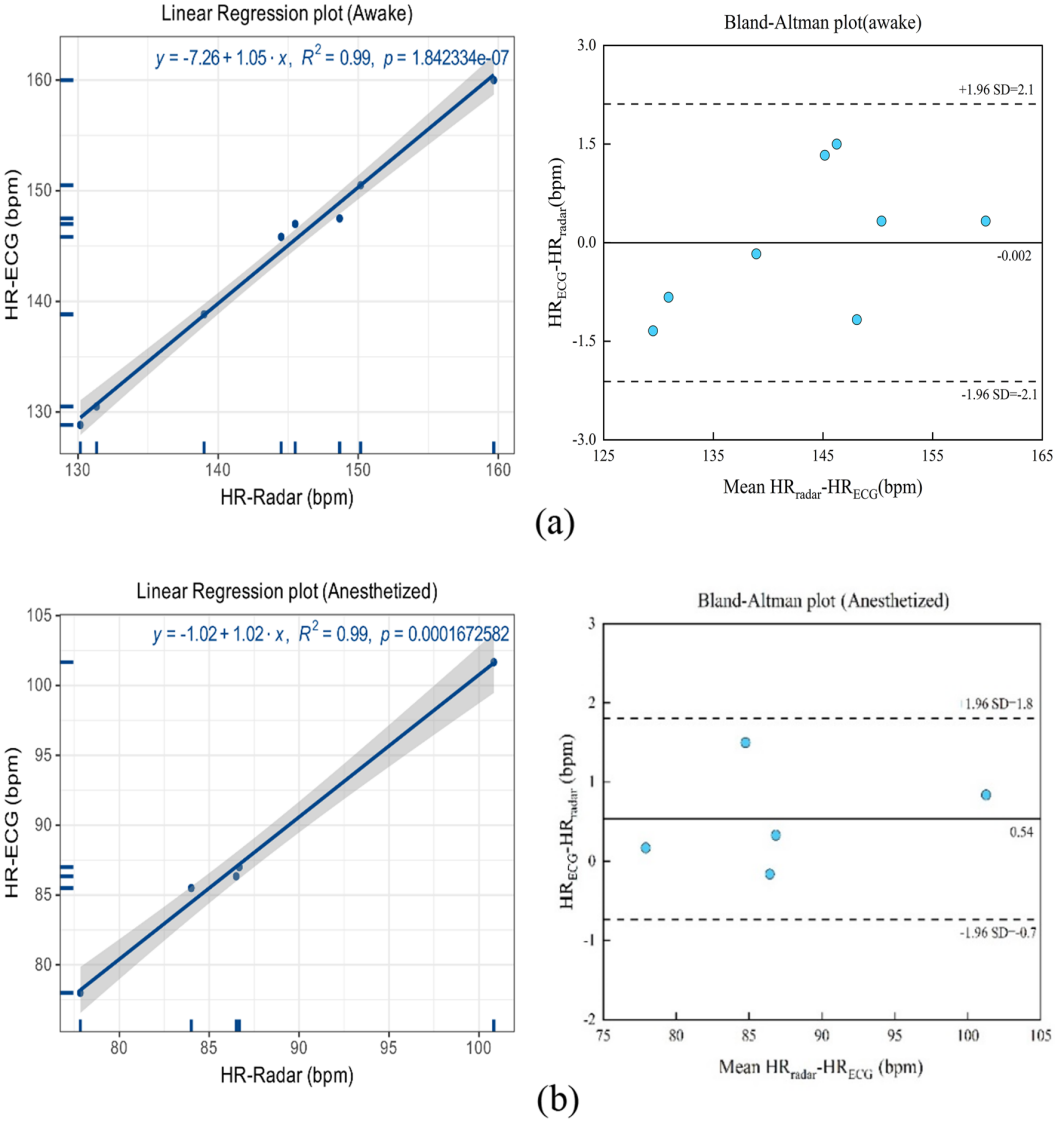
Linear regression and Bland–Altman plots comparing the HR assessed via radar and using the ECG for (**a**) awake and (**b**) anesthetized macaques.

Figure 11 shows linear regression and Bland–Altman plots comparing the HR assessed via radar and ECG for all awake and anesthetized macaques. As shown in Fig. 11a, the awake macaques exhibited a coefficient of determination of R^2^ = 0.99, and the Bland–Altman plot registered a mean difference of -0.002 bpm, with the 95% limits of agreement reaching -2.1 to 2.1 bpm. As shown in Fig. 11b, the anesthetized macaques exhibited an R^2^ of 0.99, and the Bland–Altman plot registered a mean difference of 0.54 bpm, with the 95% limits of agreement reaching -0.7 to 1.8 bpm.

The detected RR comparison results are listed in Table 4, which presents the RR estimation results for awake and anesthetized macaques with the corresponding VMD algorithm. On average, the RR obtained via ECG for awake macaques was 26.17 ± 5.21 rpm, while that for anesthetized macaques was 16.07 ± 6.26 rpm. The RR obtained via radar for awake macaques was 25.79 ± 6.72 rpm, while that for anesthetized macaques was 16.47 ± 7.11 rpm.

**Table 4:** RR average values of macaques detected via ECG and radar.

Macaque ID	State	ECG	Radar	Abs. error	Rel. error (%)
16104	Awake	38.67	41.33	2.66	6.88
152024	Awake	25.00	25.67	0.67	2.68
152030	Awake	22.17	20.00	2.17	9.79
152032	Awake	28.83	31.00	2.17	7.53
15092	Awake	26.00	23.67	2.33	8.96
151010	Awake	24.50	21.17	3.33	13.59
151007	Awake	21.67	21.33	0.34	1.57
151009	Awake	22.50	22.17	0.33	1.47
19080	Anesthetized	20.67	20.00	0.67	3.24
19092	Anesthetized	13.00	13.67	0.67	5.15
20005	Anesthetized	26.00	28.67	2.67	10.27
20042	Anesthetized	9.67	10.00	0.33	3.41
20313	Anesthetized	11.00	10.00	1.00	9.09

As shown in Fig. 10b, for the awake macaques, all RR errors remained under 4, 3, 2, and 1 rpm for 100%, 77.08%, 41.66%, and 27.08% of the measurements, respectively; while for the anesthetized macaques, the RR errors remained under 4, 2, and 1 bpm for 100%, 93.33%, and 73.33% of the measurements, respectively, which indicates a better detection success rate.

A comparison between the ECG and radar measurements showed that the mean absolute and relative errors for awake macaques were 1.75 rpm and 6.56%, respectively, while those for anesthetized macaques were 1.07 rpm and 6.23%, respectively.

**Figure 12:**
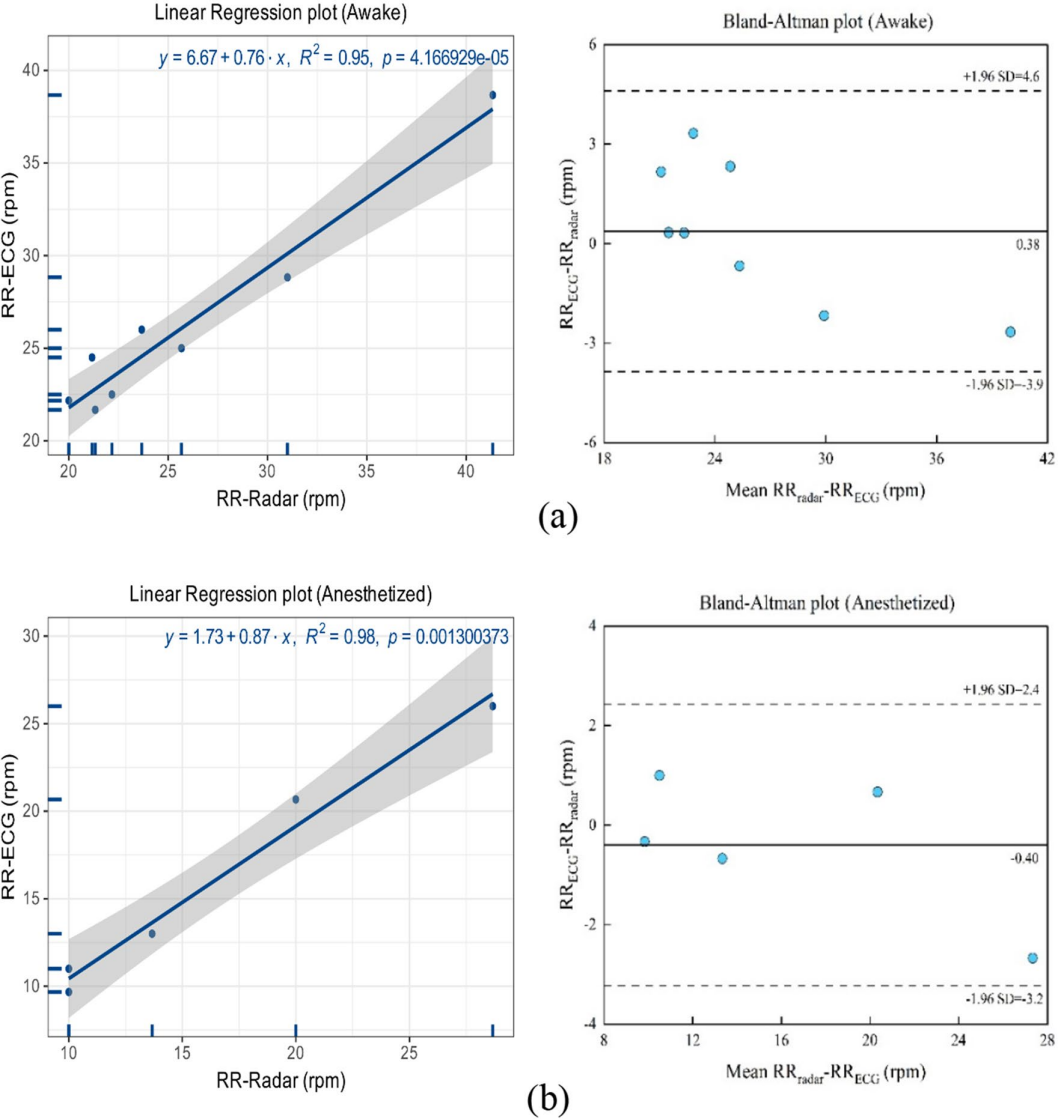
Linear regression and Bland–Altman plots comparing the RR assessed via radar and using the ECG for (**a**) awake and (**b**) anesthetized macaques.

Figure 12 shows linear regression and Bland–Altman plots comparing the RR assessed via radar and ECG for all awake and anesthetized macaques. As shown in Fig. 12a, the awake macaques exhibited an R^2^ of 0.95, while the Bland–Altman plot registered a mean difference of 0.38 rpm, with the 95% limits of agreement reaching -3.90 to 4.6 rpm. As shown in Fig. 12b, the anesthetized macaques exhibited an R^2^ of 0.98, while the Bland–Altman plot registered a mean difference of -0.40 rpm, with the 95% limits of agreement reaching -3.20 to 2.40 rpm.

It is worth noting that the HR and RR errors between the ground truth and radar measurements were less than 4 bpm and 4 rpm, respectively, in all cases considered. Thus, the radar was able to estimate the macaque HR and RR with a high accuracy.

## Discussions and conclusions

In this study, we explored the ability of a new FMCW radar system to measure the HR and RR in macaques. To the best of our knowledge, this is the first study to remotely estimate the HR and RR of captive NHPs using an FMCW radar technique, which eliminates the stress response in macaques caused by sensor contact, which in turn results in unreliable vital signs measurements.

Compared with traditional contact-based methods (e.g., ECG), the proposed contactless method was unaffected by animal hair and did not require shaving the subject before measurement. Additionally, compared implanting telemetry devices and highly invasive internal sensors for monitoring subjects in a free-moving paradigm macaque heart rate raises a range of hazards and ethical concerns, mandatory implantation might induce behavioral alterations and psychological discomfort in macaques, encroaching upon their animal welfare. Our monitoring system offers a simple and practical solution for real-time RR and HR estimation by placing the FMCW radar on the cage and facing the chest of the macaques^34–38^. As a result, there was no harm to their welfare or interference from their circadian rhythms during the experiment, and we will make the potential contributions and future applications of our experimental setup in the context of NHP research, which is crucial for experimental quality and animal welfare^39^.

Meanwhile, we verified the accuracy of the HR and RR measurements for awake and anesthetized macaques using a contact ECG as a ground truth. The experimental results indicate that the proposed FMCW radar system exhibits high accuracy, with mean absolute errors between the reference monitor and radar estimates of 0.77 bpm and 1.29 rpm for HR and RR, respectively.

Furthermore, according to the physiological characteristics of different experimental animals, after developing customizable/personalized vital signs processing algorithms, the proposed method shows promise for applications in many experimental animals for animal welfare, behavioral, neurological, and ethological research^40,41^.

Despite its many advantages, the proposed method has some limitations. The proposed method is capable of vital signs monitoring of a single stationary subject under resting, sleeping, or human commands during data capture. When the subject maintained significant movements, it was difficult for the radar to measure the respiratory and heart rates of the target because stronger motion signals can override the heartbeat and respiratory signals^42^. In this regard, more advanced vital signs processing algorithms are required for vital signs monitoring and detection of moving subject^31,43^ or of multiple static targets situated at different range bins^44^. These aspects will be further explored in future work, making the proposed method highly practical under real-world scenarios.

## Data Availability

The data used to support this research are available from the corresponding author on request.
